# A causal relationship between right paraduodenal hernia and superior mesenteric artery syndrome: a case report

**DOI:** 10.1186/1752-1947-4-159

**Published:** 2010-05-27

**Authors:** Tadaomi Fukada, Hideyasu Mukai, Fumihiko Shimamura, Takeshi Furukawa, Masaru Miyazaki

**Affiliations:** 1 Department of General Surgery, Chiba Emergency Medical Center, Isobe, Mihama-ku, Chiba, 260-0856, Japan; 2 Department of General Surgery, Graduate School of Medicine, Chiba University, Inohana Chuo-ku, Chiba, 260-8677, Japan

## Abstract

**Introduction:**

Cases of right paraduodenal hernia and superior mesenteric artery syndrome have been reported separately, but their occurrence in combination has not been reported.

**Case presentation:**

A 46-year-old Japanese man who had never undergone laparotomy was admitted to our hospital due to an acute abdomen. An enhanced multidetector-row computed tomography scan of our patient showed a cluster of small intestines with ischemic change in his right lateral abdominal cavity. Emergency surgery was subsequently performed, and strangulation of the distal jejunum along with incidental right paraduodenal hernia was found. His necrotic ileum was resected, and the jejunum encapsulated by the sac was repaired manually without reduction.

Three days after the operation, however, our patient developed vomiting. An upper gastrointestinal series revealed a straight line cut-off sign on the third portion of his duodenum. A second enhanced multidetector-row computed tomography scan showed that he had a lower aortomesenteric angle and a shorter aortomesenteric distance compared to his condition before his right paraduodenal hernia was surgically repaired. We strongly suspected that the right paraduodenal hernia repair may have induced superior mesenteric artery syndrome. On the 21st post-operative day, duodenojejunostomy was performed because conservative management had failed.

**Conclusions:**

In this case, enhanced multidetector-row computed tomography, which permits reconstructed multiplanar imaging, helped us to visually identify these diseases easily. It is important to recognize that surgical repair of a right paraduodenal hernia may cause superior mesenteric artery syndrome.

## Introduction

Paraduodenal hernia was first described in 1857, while superior mesenteric artery syndrome (SMAS) was first defined in 1861 [1]. While the occurrence of each case has been reported separately, their simultaneous occurrence and the possibility of their causal relationship have never been reported, to our knowledge. So far, there have been some reports on the use of ultrasound, upper gastrointestinal contrast studies, and computed tomography (CT) for the diagnosis of these diseases. However, the abilities of these modalities to diagnose these conditions are insufficiently discussed, and delayed and incorrect diagnosis has been fatal in some cases. A rare case of surgical repair of right paraduodenal hernia (RPH) that may have induced SMAS is reported, and the usefulness of enhanced multidetector-row computed tomography (MDCT) for the diagnosis of these diseases is highlighted. Recently, enhanced MDCT, which can provide reconstructed multiplanar imaging, has become a substitute for angiography and magnetic resonance imaging (MRI) because of its quality and convenience. MDCT allowed for easy visual identification of these diseases.

## Case presentation

A slim 46-year-old Japanese man was brought to the emergency room of the Chiba Emergency Medical Center because of intermittent epigastric pain. He had no history of abdominal surgery. His initial blood and urine examinations showed no abnormalities. However, the pain had been getting worse and signs of peritoneal irritation appeared within a few hours.

**Figure 1:**
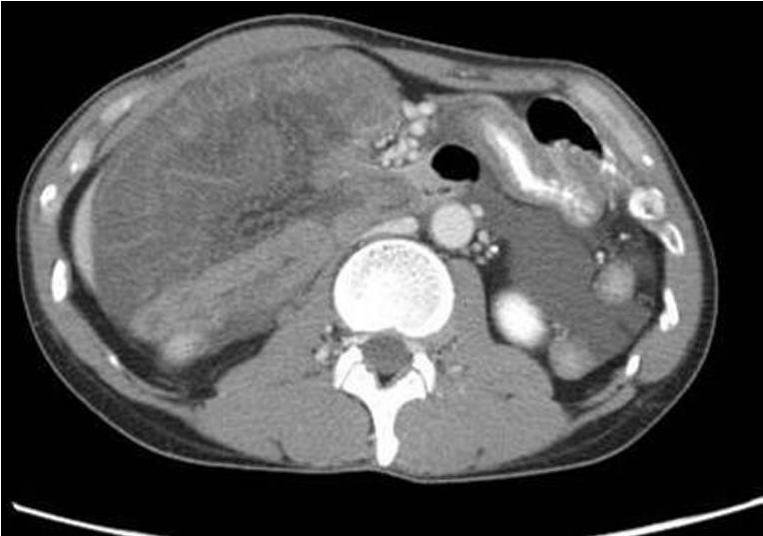
**Pre-operative enhanced abdominal multidetector-row computed tomography shows intestinal cluster in the right lateral abdominal cavity**. The intestinal wall is edematous and not well-enhanced.

An abdominal enhanced MDCT scan without multiplanar reconstruction was then performed, which revealed small bowel loops with suspicious ischemic change in the right lateral part of the abdomen of our patient (Figure 1). An internal hernia with strangulation was diagnosed, and emergency surgery was subsequently performed.

**Figure 2:**
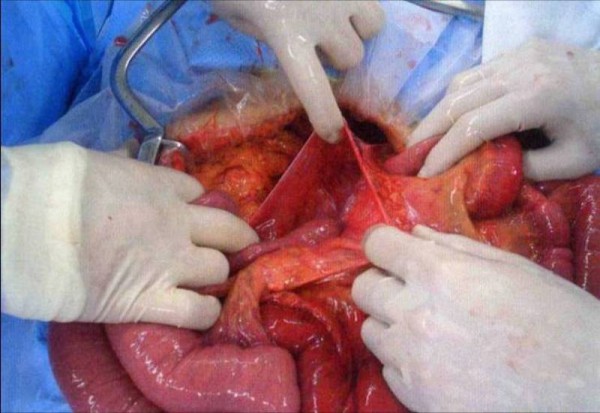
Operative findings show the unfixed hernia orifice by the side of the ligament of Treitz.

During surgery, a band between the mesentery near the cecum and the retroperitoneum was found. Part of his jejunum had been sliding under the band and had thus become necrotic. The necrotic bowel, which was 50 cm in length, was resected. Furthermore, one-quarter of the proximal small intestine was encapsulated in a peritoneal sac that was surrounded by transverse mesocolon. The bowel was entering the Waldeyer's fossa through an opening beside the ligament of Treitz (Figure 2). A right paraduodenal hernia was then diagnosed.

The bowel was removed manually from the sac. There was no sign of ischemic change. The fossa was approximated tightly with no free space. Furthermore, there was no malrotation of our patient's bowel, and the cecum was located in the normal position without Ladd's band. On the third post-operative day, our patient complained of nausea and he started vomiting, thus a nasogastric tube was placed. After that, more than 2000 mL of bilious gastric juice was drained from him every day.

**Figure 3:**
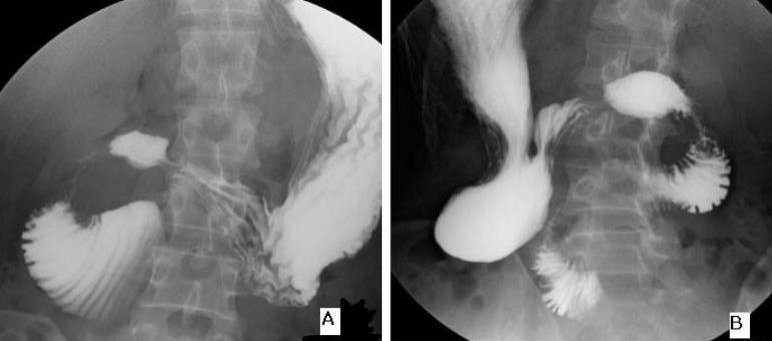
**Upper gastrointestinal series shows: ** (A) Upper gastrointestinal series shows stricture of the duodenal third portion with a straight line cut-off sign in supine position. (B) In the prone position, the contrast medium passes through the obstructed part to the distal side of duodenal third portion.

An upper gastrointestinal contrast series revealed dilatation of the right side of the duodenal third portion with a straight line cut-off sign. The contrast medium did not pass to the proximal side in supine position, although it did pass with postural change to the prone position (Figure 3). SMAS was strongly suspected, and an enhanced MDCT of our patient was again performed to examine the stricture around his duodenum and SMA. A multiplanar reconstruction of these was performed, and pre-operative images were also taken.

**Figure 4:**
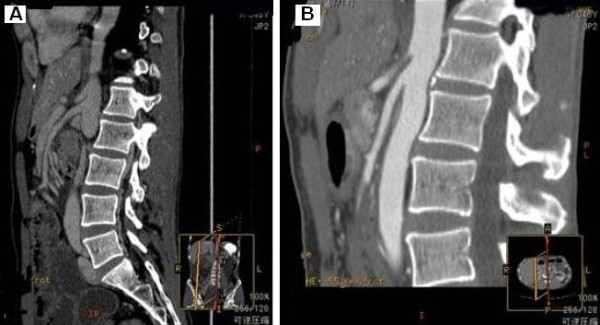
**Reconstructed enhanced multidetector-row computed tomography reveals the following:(A) **before the surgical repair of the right paraduodenal hernia, the aorto-mesenteric angle is 44°, and the aorto-mesenteric distance is 28 mm. There are intestinal gas bubbles under the aorto-mesenteric junction. **(B) **After the repair, the aorto-mesenteric angle has narrowed to 14°, and the aorto-mesenteric distance has shortened to 5 mm. Furthermore, no intestinal gas can be seen under the angle.

The images revealed a very narrow aortomesenteric angle (AMA) and a short aortomesenteric distance (AMD) (Figure 4A). These data were compared with the pre-operative findings. The AMA had changed from 44 to 14 degrees, and the AMD had changed from 28 mm to 5 mm. Furthermore, before the operation, bowel was present under our patient's aorto-SMA junction, but the bowel disappeared after surgery (Figure 4B). Thus, SMAS caused by the surgical repair of RPH was diagnosed.

On the 21st post-operative day, because the symptoms had not improved with conservative management, a duodenojejunostomy (DJ), which left a duodenal loop, was performed on our patient.

**Figure 5:**
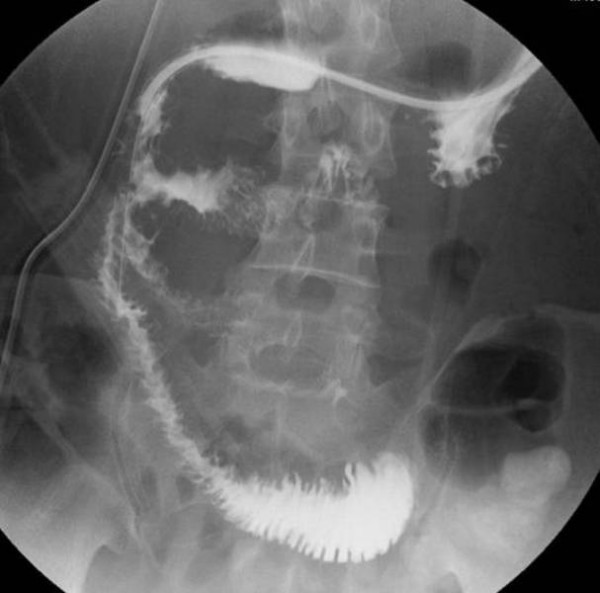
**Post-operative upper gastrointestinal series shows good passage through the anastomosis**.

Our patient's post-operative course was uneventful. The post-operative upper gastrointestinal series showed good passage through the anastomosis (Figure 5).

## Discussion

Paraduodenal hernia and SMAS are both relatively rare and can cause intestinal obstruction. Paraduodenal hernia was defined first by Treitz in 1857 as a duodenojejunal fossa, and SMAS was defined by Von Rokitansky in 1861 as duodenal dilatation and obstruction due to compression of the third portion of the duodenum by the superior mesenteric artery [1].

Paraduodenal hernia is an internal hernia that accounts for 0.6% to 5.8% of small intestinal obstructions [2]. Internal hernias can be either congenital or acquired. Congenital types are paraduodenal (53%), transmesenteric (12%), at the foramen of Winslow (8%), paracecal (6%), and transomental (<5%) [3].

Paraduodenal hernia occurs three times more often in men [4-6]. It has two main types, left-sided and right-sided, with the left-sided type accounting for 75% of reported cases [2].

An RPH represents a hernial sac entrapping the small intestine through the Waldeyer's fossa. The fossa is the first part of the mesentery of the jejunum, and is located inferior to the third portion of the duodenum and behind the superior mesenteric artery (SMA). This specific location is very important, because the bowel cluster that passes through the hernia orifice into the sac seems to lift up the SMA to a ventral position. As a result, the angle of the SMA to the aorta, which is the AMA, is probably wider than in the normal state. This is the reason why the surgical repair of RPH may have caused SMAS.

Using the keywords RPH and SMAS, more than 60 clinical cases of RPH were identified on PubMed, and none discussed the relationship between RPH and SMAS [7].

The symptoms of RPH are variable and non-specific, such as chronic dyspepsia, intermittent colicky abdominal pain, and vomiting [8]. Furthermore, in many RPH cases, malrotation of the digestive tract has been detected. On the other hand, the symptoms of SMAS are specific, including nausea, bilious vomiting, epigastric pain (with normal or hyperactive bowel sounds), and postprandial abdominal fullness and distension. The vomiting decompresses the stomach and produces asymptomatic intervals that last for several hours before the next episode.

The treatment of RPH is generally surgical. The procedure involves separating the sac and placing the intestines in the correct position. During this procedure, it is important to avoid injuring the mesenteric vessels. Furthermore, in children, appendectomy should also be performed.

On the other hand, the initial management of SMAS is conservative, but if conservative management fails, surgical management should be immediately considered because SMAS can be fatal due to acute metabolic alkalosis and dehydration [9]. The common surgical procedure for SMAS is DJ.

With our patient, the initial complaint worsened from mild epigastralgia to uncontrollable general abdominal pain. Enhanced MDCT, which did not initially include reconstructed multiplanar images, showed ischemic change of the bowel in our patient's right abdominal cavity. A strangulated ileus due to internal hernia was highly suspected, but paraduodenal hernia was not diagnosed.

When we performed emergency surgery, we found a gangrenous change in our patient's ileum due to a cord that was located in his right lower retroperitoneum, and RPH was incidentally identified. The ischemic bowel was resected, and the jejunum sliding into his hernia sac was worked out manually without reduction.

During surgery, there was no evidence of malrotation, including Ladd's band. However, on the third post-operative day, our patient developed abdominal distension, nausea and vomiting, and 2000 mL of bilious gastric contents was drained from his nasogastric tube daily.

An upper gastrointestinal barium contrast study showed stricture of the third portion of his duodenum with a straight line cut-off sign in supine position, although, in the prone position, contrast medium passed smoothly through the obstructed part. As this is one of the criteria of SMAS, we highly suspected SMAS. To evaluate his abdomen, including the vessels, we again performed an enhanced MDCT and the images, along with the pre-operative images, were reconstructed for multiplanar imaging.

**Figure 6:**
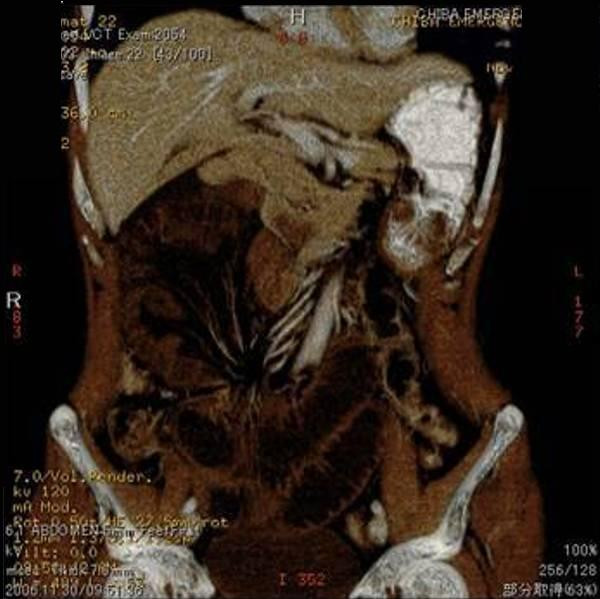
**Retrospective multiplanar reconstructed enhanced multidetector-row computed tomography images reveal an abnormal cluster of digestive loops in the right lateral abdominal cavity and mesenteric vessel changes such as twisting and stretching**.

These three-dimensional images enabled us to diagnose RPH and SMAS more easily. A review of the pre-operative reconstructed MDCT images revealed an abnormally located cluster of digestive loops with bowel obstruction and mesenteric vascular changes, such as twisting and stretching (Figure 6). Mesenteric vascular abnormalities on imaging are crucial for the diagnosis of internal hernia.

Furthermore, pre-operative and post-operative images showed obvious visual changes in the aorto-SMA junction. Before surgery, the AMA was wide, and some intestinal gas was seen under the angle. However, post-operatively the angle had become narrow, and the gas could not be identified (Figures 4A and 4B)

There have been some reports of MDCT imaging findings of paraduodenal hernia and SMAS [7,10-15]. However, enhanced MDCT images of these diseases have not been previously done, which is one reason why in many cases of suspected internal hernia, emergency surgery was performed before a correct diagnosis could be made. Because a delay in diagnosis could be fatal in both diseases [9,16,17] the mortality rate of gangrenous cases of RPH is reported to be above 20% [16,17].

In our case, pre-operative MDCT showed a wide AMA, a long AMD, and several intestinal gas bubbles under the angle. However, the post-operative enhanced MDCT revealed a narrow AMA, a short AMD, and the absence of bowel gas. We hypothesized that our patient had been suffering from RPH without complete bowel and vessel obstruction, so that the hernia cluster had kept the AMA wide. However, the repair of the hernia might have caused the AMA to narrow and squeeze the duodenal third portion, thus causing SMAS. In this case, reconstructed multiplanar images of enhanced MDCT allowed us to easily visualize and identify these diseases.

Recently, laparoscopic repair of RPH and SMAS has been reported as a minimally invasive alternative; an initial laparoscopic approach could be appropriate for both the diagnosis and treatment of suspicious cases.

## Conclusions

RPH is a rare cause of small bowel obstruction and a diagnostic challenge. Reconstructed enhanced MDCT images are very useful to diagnose it. Furthermore, it is important to recognize that repairing RPH may induce SMAS. Especially in cases with vomiting after surgical repair of RPH, SMAS should be considered in the differential diagnosis.

## Abbreviations

AMA: aorto-mesenteric angle; AMD: aorto-mesenteric distance; CT: computed tomography; DJ: duodenojejunostomy; MDCT: multidetector-row computed tomography; RPH: right paraduodenal hernia; SMAS: superior mesenteric artery syndrome.

## Competing interests

The authors declare that they have no competing interests.

## Authors' contributions

TF was a major contributor in writing the manuscript and in interpreting the data regarding the operation and outcomes. TF and FS gathered and analyzed the data regarding the history and the operative management of our patient. HM provided clinical insights and final approval for the manuscript as the head of the department. All authors read and approved the final manuscript.

## Consent

Written informed consent was obtained from our patient for publication of this case report and any accompanying images. A copy of the written consent is available for review by the Editor-in-Chief of this journal.
